# Omics for deciphering oral microecology

**DOI:** 10.1038/s41368-023-00264-x

**Published:** 2024-01-09

**Authors:** Yongwang Lin, Xiaoyue Liang, Zhengyi Li, Tao Gong, Biao Ren, Yuqing Li, Xian Peng

**Affiliations:** State Key Laboratory of Oral Diseases & National Center for Stomatology & National Clinical Research Center for Oral Diseases, West China Hospital of Stomatology, Sichuan University, Chengdu, China

**Keywords:** Oral diseases, Microbial ecology, Microbiology techniques, Microbiome

## Abstract

The human oral microbiome harbors one of the most diverse microbial communities in the human body, playing critical roles in oral and systemic health. Recent technological innovations are propelling the characterization and manipulation of oral microbiota. High-throughput sequencing enables comprehensive taxonomic and functional profiling of oral microbiomes. New long-read platforms improve genome assembly from complex samples. Single-cell genomics provides insights into uncultured taxa. Advanced imaging modalities including fluorescence, mass spectrometry, and Raman spectroscopy have enabled the visualization of the spatial organization and interactions of oral microbes with increasing resolution. Fluorescence techniques link phylogenetic identity with localization. Mass spectrometry imaging reveals metabolic niches and activities while Raman spectroscopy generates rapid biomolecular fingerprints for classification. Culturomics facilitates the isolation and cultivation of novel fastidious oral taxa using high-throughput approaches. Ongoing integration of these technologies holds the promise of transforming our understanding of oral microbiome assembly, gene expression, metabolites, microenvironments, virulence mechanisms, and microbe-host interfaces in the context of health and disease. However, significant knowledge gaps persist regarding community origins, developmental trajectories, homeostasis versus dysbiosis triggers, functional biomarkers, and strategies to deliberately reshape the oral microbiome for therapeutic benefit. The convergence of sequencing, imaging, cultureomics, synthetic systems, and biomimetic models will provide unprecedented insights into the oral microbiome and offer opportunities to predict, prevent, diagnose, and treat associated oral diseases.

## Introduction

The oral cavity serves as a vital interface for the exchange of substances between the human body and the external environment. As one of the most diverse microbial communities in the human body, the oral microbiome harbors over 700 bacterial species adhering to oral mucosa and dental surfaces.^1^ The oral microbiome encompasses these unique microbial communities, consisting of bacteria, fungi, viruses, archaea, and protozoa, as well as their collective genomes and surrounding microenvironment.^2^ Maintaining a balanced microbial composition is imperative for oral and systemic health, while dysbiosis can precipitate disease. The dysbiosis of oral microecology refers to ecological instability arising from factors such as inflammation, poor oral hygiene, dietary habits, or host genetics,^3^ and which has been implicated in common conditions like periodontal diseases and dental caries. Moreover, aberrant host-oral microbiome interactions can have systemic implications in diabetes, cardiovascular disease, rheumatoid arthritis, respiratory disease, colorectal cancer, inflammatory bowel disease, and Alzheimer’s disease.^3,4^ Consequently, comprehensive analysis of oral microbiome structure and function is essential for elucidating principles governing community assembly, microecology regulation, and disease etiology.^5^ Historically, oral microbiome research relied upon techniques like microbial isolation, pure culture, and fingerprinting.^6^ However, these methods had limited utility for holistic characterization due to technical constraints. The advent of high-throughput sequencing and mass spectrometry has revolutionized oral microbiome research, enabling in-depth interrogation of ecological structure and function.^7,8^

**Fig. 1:**
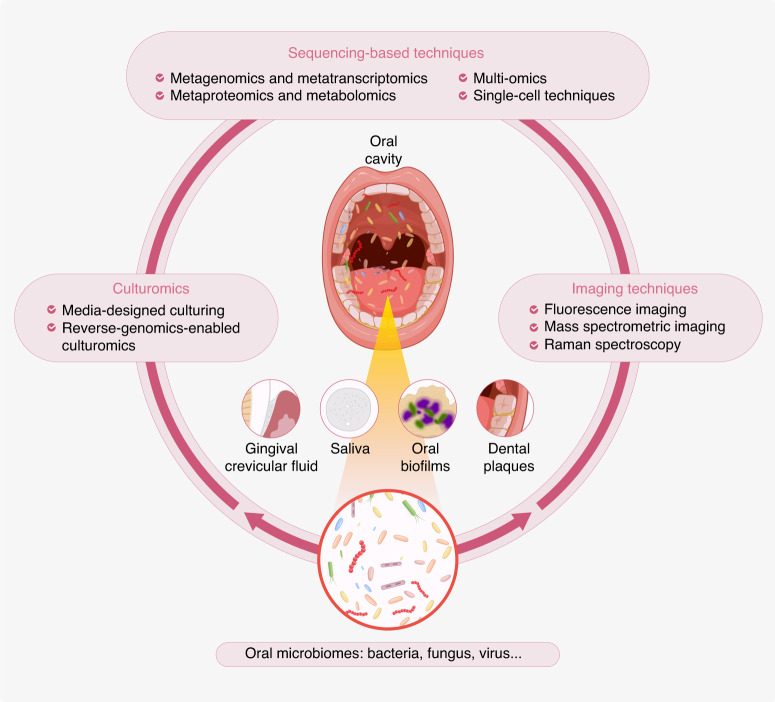
Outline of strategies for deciphering oral microecology. The oral microbiome encompasses a diverse community of microorganisms residing in the oral cavity, including saliva, teeth, gums, tongue, and other oral surfaces. This intricate ecosystem consists of various species of bacteria, viruses, fungi, and other microbes. Oral microecology is crucial for maintaining both oral and overall systemic health. The strategies for deciphering oral microecology can be categorized into sequencing-based technologies, imaging techniques, and cultureomics

Nevertheless, key challenges remain. Low abundance taxa are difficult to isolate or culture in vitro, with underrepresented genomes in metagenomic samples.^9^ While descriptive cataloging of oral microbiome structure and diversity has matured, transitioning from quantity to quality and structure to function remains imperative for microbiome research.^10^ To overcome these hurdles, promising technologies have emerged including sequencing, microbiome imaging, and cultureomics^11,12^ (Fig. 1). These innovations offer opportunities to enrich the understanding of oral microecology and its contributions to health and disease (Table 1).

**Table 1:** Studies of oral microecology-related technology

Year	Samples from	Techniques	Results	Ref.
2018	Plaque samples from elderly individuals of 24 caries patients and 22 healthy controls.	Metagenome	A total of 16 phyla, 29 classes, 49 orders, 79 families, 149 genera, and 305 species from all the specimens were identified. Biomarkers associated with caries and health were identified.	^16^
2021	Salivary samples from 23 children with caries and 24 children with healthy dentition.	Metagenome	150 bacterial species, including 42 novel taxa have been identified, which include the Saccharibacteria clades G3 and G6.	^94^
2019	Salivary samples from preschoolers, 25 with severe early childhood caries, and 19 age-matched, caries-free children as controls.	Metagenome	26 264 differentially abundant genes have been identified and clustered into 18 metagenomic species, with 12 enriched in the ECC group and 6 enriched in the healthy control.	^18^
2018	Biofilm samples from 10 children with caries and 4 children without caries.	Metatranscriptomes	Gene expression sequences mapped into 164 taxa that comprised 108 named species, 12 phylotypes, 33 bacterial genus-level identifications, and 13 viruses.	^20^
2018	Subgingival plaque and gingival crevicular fluid samples from 10 individuals undergoing an experimental gingivitis treatment.	16S v4-v5 rRNA amplicon and metatranscriptome sequencing	Many virulence-related transcripts were significantly differentially expressed in individual and across pooled patient samples during the early stages of transition to gingivitis.	^22^
2021	Subgingival plaque samples of healthy, gingivitis, and periodontitis sites in the same oral cavity were collected from 21 patients.	Metatranscriptomics and network analyses	At the species level, the rc-rRNAs were assigned to 225 bacterial taxa; differences in bacterial compositions and gene expression profiles among the three statuses were elucidated.	^24^
2016	Saliva samples were collected from 10 patients with periodontitis, 10 patients with dental caries, and 10 orally healthy individuals.	Liquid chromatography/tandem mass spectrometry proteomic approach	1 946 bacterial proteins and 2 090 human proteins were identified, and the human protein profiles displayed significant overexpression of the complement system and inflammatory markers in periodontitis and dental caries.	^27^
2015	Salivary samples from 168 subjects with 5 categories of periodontal status.	Liquid chromatography-mass spectrometry and gas chromatography-mass spectrometry	272 microbial species were grouped into 8 distinct microbial communities, and their correlation to periodontal disease status and salivary metabolome composition have been explored.	^29^
2019	Supragingival plaque in response to the use of arginine or fluoride toothpaste from 83 adults of different caries status.	Metabolomic analyses were performed using a Thermo Q-Exactive high-resolution mass spectrometer	The metabolic profiles of plaque treated with arginine significantly affected the concentrations of 16 metabolites, while fluoride affected the concentrations of 9 metabolites.	^31^
2017	Sub- and supragingival biofilms in adults with chronic periodontitis before and after treatment with 0.25% sodium hypochlorite.	Microbial 16S rRNA amplicon sequencing, shotgun metagenomics, and tandem mass spectrometry	It has been found that markedly higher taxonomic instability in patients who did not improve posttreatment than in patients who did improve while the opposite pattern occurred in the metabolic profiles.	^33^
2021	Supragingival plaque samples from 97 study participants with periodontal disease and/or diabetes.	16S rDNA sequencing, metabolomics, lipidomics, and proteomics analyses	Researchers have identified 2 790 operational taxonomic units and 2 025 microbial and host proteins per sample and quantified 110 metabolites and 415 lipids; and significant connections between host-derived proteins and periodontal disease have also been demonstrated.	^34^
2019	Human oral microbiota sample.	Bacterial labeling with antibiotic-based probes	Gram-negative and Gram-positive bacteria were distinctly stained and showed dramatic phenotypical diversities.	^50^
2021	Plaque biofilms collected at 7 timepoints from a healthy donor for 27 months.	High Phylogenetic Resolution microbiome mapping by Fluorescence in situ Hybridization	The technology consists of 233 probes that target 54 bacterial genera and observed corn-cob-like structures of plaque biofilm.	^56^
2021	*P. gingivalis*, *A. actinomycetemcomitans*, cultivated in human saliva.	Surface-enhanced Raman spectroscopy	It is possible to detect and identify *P. gingivalis* and *A. actinomycetemcomitans* strains in human saliva.	^89^
2020	The peri-implant and sub-gingival plaques of 4 patients.	Culturomics	Out of 48 isolated species, only 30 were previously identified, while 12 species had no prior association with the oral cavity, and 5 of them were never isolated from clinical samples.	^92^
2019	Human oral samples from healthy donors or individuals with periodontitis.	Single-cell genomic, Reverse-genomics-enabled cultivation of microorganisms	Three different species-level lineages of human oral *Saccharibacteria* (TM7). and human oral SR1 bacteria have been isolated and cultivated.	^6^
2020	Sub- and supra-gingival samples were pelleted and inoculated into broth cultures of potential bacterial host cells.	Culturomics and 16S rRNA sequencing	Thirty-two isolates representing four species of *Saccharibacteria* were isolated in stable coculture with three species of host bacteria from the phylum *Actinobacteria*.	^95^

## Sequencing-based techniques

Our understanding of oral microbial ecology has expanded substantially aided by resources such as the Human Oral Microbiome Database (HOMD).^13^ HOMD serves as a meticulously curated and comprehensive platform that houses genomic and phylogenetic data on the human oral microbiome, encompassing both 16S rRNA sequencing results and metagenomic profiling. The recently expanded eHOMD^13^ now encompasses the genetic information on ~771 microbial species and strains, with 687 from the oral cavity and 84 from aerodigestive regions beyond the mouth.

**Fig. 2:**
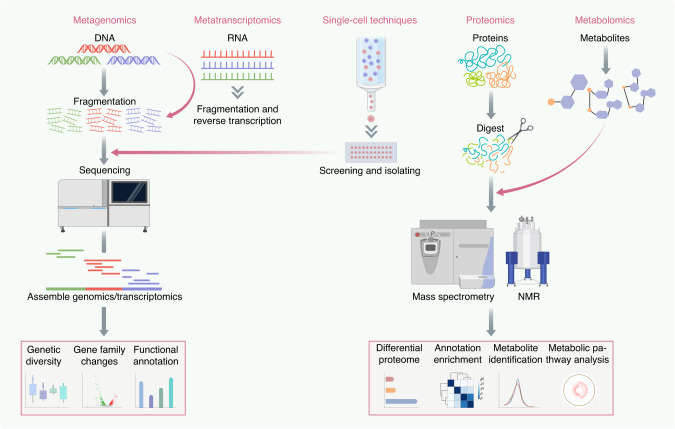
Sequencing-based technologies. Sequencing-based technology provides comprehensive molecular profiling of the oral microbiome. These advanced techniques include metagenomics, metatranscriptomics, metaproteomics, metabolomics platforms, and single-cell techniques. Metagenomics involves the fragmentation of DNA in a sample, allowing for the analysis of the entire microbial community. Metatranscriptomics focuses on the analysis of mRNA after extraction, providing insights into gene expression patterns. The resulting sequences can be aligned to reference genomes or transcriptomes to identify the microbial community and differentially expressed genes within the microbiome. Metaproteomics and metabolomics are employed to quantify the proteins and metabolites produced by the microbiome, respectively. Techniques such as mass spectrometry and nuclear magnetic resonance spectroscopy are commonly used for accurate quantification, enabling the functional characterization of proteins and metabolic pathways within the microbial community. Single-cell techniques enable the analysis and characterization of individual cells that have been sorted and isolated from a heterogeneous population. This is achieved through whole genome amplification and subsequent sequencing, allowing for detailed examination of genetic and functional traits at the single-cell level

While 16S/18S rRNA sequencing is frequently utilized to estimate microbial population size and diversity, this method generally only allows for distinction to the genus level and provides limited functional insights. More recently, metagenomic and metatranscriptomic approaches have provided greater evidence regarding microbial functional potential—which genes are present and/or expressed within the population.^14^ Metaproteomics and metabolomics serve as two contemporary fields that have significantly advanced our understanding of how oral communities change functionally and taxonomically in health and disease, as well as in response to changing environmental conditions (Fig. 2).

### Metagenomics and metatranscriptomics

Metagenomics involves analyzing the genomic content of microbial communities, while metatranscriptomics focuses on capturing the total messenger RNA (mRNA) content of a sample to explore the functional activity of the microbiome.^15^ These approaches have provided valuable insights into the taxonomic composition, functional potential, and gene expression profiles of the oral microbiome. Which have enhanced our understanding of oral health and disease, and have the potential to guide the development of new interventions.^15^

Metagenomic analysis has been used to study the bacterial composition of the oral microbiome in various conditions. For example, a study on elderly individuals with caries detected a total of 149 genera, and 305 species from oral specimens.^16^ The study found that the bacterial composition of caries-active and caries-free groups was similar, however, differences in the relative abundances of specific bacteria were observed. Functional predictions based on metagenomic data indicated active microbial metabolism in the oral bacterial community. Biomarkers associated with caries and health were identified, and the functional prediction of the microbiota was performed. These findings contribute to the understanding of the microbiological causes of caries in elderly individuals and may lead to the development of new prevention and treatment methods. Samples from children with or without caries suggested significant differences in the metagenomic analysis of the microbiota.^17^ *Rothia*, *Neisseria*, and *Haemophilus* spp. were associated with good dental health, while *Prevotella* spp, *Streptococcus mutans*, and *Epstein-Barr* virus were more prevalent in children with caries.^16^ Functional examination showed extensive alterations in metabolic pathways, including enriched functions in sugar metabolism, in the severe early childhood caries group, indicating that some of these can potentially be exploited as therapeutic targets or diagnostic markers.^18^

Metatranscriptomic analysis, on the other hand, provides insights into the gene expression profiles of the oral microbiome. By capturing the total mRNA content, metatranscriptomics allows for the exploration of functional activity in different conditions, such as disease versus health or different diets.^19^ For example, a study comparing diseased and healthy oral communities found enhanced diversity in taxonomy and gene expression over disease severity.^20^ Caries-associated communities exhibited diminished activity of *Streptococcus* and increased activity of *Lactobacillus*, as well as elevated levels of urease and arginine deiminase production.^20^ In contrast, caries-free communities expressed high levels of alcohol dehydrogenase, which may counteract bacterial acid production.^20,21^ Similarly, the progression from gingivitis to periodontitis involves shifts in function and community composition,^22^ with upregulation of processes associated with virulence in gingivitis, and increased activities associated with lysine degradation as well as butyrate and pyruvate metabolism in periodontitis.^23^ Gradual increases in transcriptome differences are observed from health to gingivitis and ultimately to periodontitis.^24^

### Metaproteomics and metabolomics

Metaproteomics involves cataloging the abundance of microbial proteins in a sample, providing insights into the functional activity of the microbiome. This is typically done through protein extraction and tandem mass spectrometry analysis.^25^ On the other hand, metabolomics focuses on identifying the small molecules produced by the microbiome, which can serve as indicators of health or dysbiosis. Metabolites are quantified using techniques such as chromatography, mass spectrometry, and nuclear magnetic resonance.^26^ These approaches also have the potential to provide insights into disease mechanisms and identify potential biomarkers.

Compared with the study of human salivary proteome, the salivary proteome of oral microorganisms is limited. Belstrøm et al. have characterized salivary metaproteomes in healthy individuals and patients with dental caries or periodontitis. They identified a total of 4161 proteins, including both human and bacterial proteins. The bacterial proteins originated from 20 different genera and 81 different species, with *Streptococcus*, *Prevotella*, *Veillonella*, *Rothia*, and *Neisseria* being the most abundant genera.^27^ The bacterial portion of the metaproteome may be inadequate for biomarker analysis of periodontitis and caries, while certain human proteins show promise as potential future biomarkers for oral disease status. Belda-Ferre et al. conducted a metaproteomic study to profile the oral biofilms of healthy and caries-affected patients.^28^ They detected a total of 7771 bacterial proteins and provided valuable insights into the protein composition of clinical oral biofilms. The majority of bacterial proteins identified were associated with central metabolic and housekeeping processes. The study also identified potential biomarkers that could differentiate between healthy and diseased individuals, offering the possibility of identifying caries-prone individuals before the formation of caries lesions.

Metabolomic studies have been instrumental in uncovering the metabolites produced by oral microbiota. For example, Marchesan et al. conducted research on the salivary metabolome before and after experimental biofilm overgrowth, and observed a correlation between cyclodipeptides and more severe periodontal disease.^29^ Similarly, volatile metabolites have been detected in bacterial cultures and saliva samples, and unique clusters of metabolites and specific bacteria associated with periodontitis have been identified.^30^ When it refers to plaque biofilms, Nascimento et al.’ research focused on the effects of arginine and fluoride-containing toothpaste on dental plaque samples.^31^ By identifying specific metabolite features, they detected differences in phenylalanine and agmatine levels between caries-free and caries-active sites. This suggests that these metabolites may have a potential influence on the oral microbiota.

### Multi-omics

The emergence of various sequencing technologies has led to a growing trend in research to integrate multiple omics approaches for a comprehensive investigation of the oral microbiome. This integration, known as multi-omics, provides a holistic understanding of taxonomic composition, functional potential, gene expression profiles, and metabolic activities in the oral microbiome, thus offering a detailed view of the complex interactions within the oral microecology, leading to new insights into oral health and disease.^32^ For example, Califf et al. employed a multi-omics approach combining 16S rRNA amplicon sequencing, shotgun metagenomics, and tandem mass spectrometry analysis to analyze biofilms in patients with chronic periodontitis before and after treatment.^33^ Their findings suggest that this multi-omics approach, particularly metabolomics analysis, can improve treatment prediction and identify patients who are likely to respond positively to treatment. Another study by Overmyer et al. is the integration of metagenomics, metabolomics, lipidomics, and proteomics analyses to study the oral microbiome concerning conditions like prediabetes, type 2 diabetes, and periodontal diseases.^34^ This comprehensive approach identified microbial and host proteins, metabolites, and lipids in dental plaque samples from patients with these conditions. The study revealed higher levels of *Fusobacterium* and *Tannerella* in pre-diabetes/diabetes patients and elevated levels of specific lipids and host proteins associated with actin filament rearrangement in periodontal disease patients. The cross-omic correlation analysis also identified associations between *Lautropia* and a rare lipid compound.

### Single-cell techniques

Single-cell sequencing is an emerging field that holds great potential for advancing our understanding of the oral microbiome. This technique involves isolating and sequencing the genomes, transcriptomes, and proteomes of individual microbial cells, enabling the study of low-abundance and less-prevalent bacteria.^6^ Moreover, by isolating cells based on phylogenetic markers or physiological characteristics, single-cell sequencing enables targeted investigations of individual cell behaviors within complex microbial communities regardless of their abundance.^35^ Isolation techniques, such as flow cytometry or microfluidic separation, are employed to obtain single cells for analysis.^36^

This approach has been particularly useful in characterizing challenging-to-cultivate taxonomic lineages within the oral microbiota, such as candidate bacterial phyla TM7, SR1, as well as oral *Chloroflexi.*^2^ The single-cell TM7 genomes generated by Marcy et al. have served as a foundation for the labeling, isolating, sequencing, and cultivating of additional members of this oral lineage.^6^ Similarly, single-cell genomic investigation of SR1 bacteria has revealed a unique genetic recoding involving the opal stop codon.^37^ Furthermore, single-cell sequencing has provided insights into various *Bacteroidetes* species associated with oral health or disease, aiding in the identification of health-disease markers. For example, *Tannerella* sp. BU063, found in healthy oral communities, lacks virulence genes, while *Tannerella* sp. BU045, associated with periodontitis, has had multiple single-cell genomes sequenced, aiding in the identification of health-disease markers.^38^ Additionally, this technique has enabled the characterization and isolation of previously unknown oral *Deltaproteobacteria* genera enriched in periodontitis, shedding light on their genomic characteristics and pathogenic properties.^39^ These studies exemplify the power of single-cell sequencing in unraveling the genomic profiles and ecological roles of previously uncultured oral microorganisms.^40^

## Oral microflora imaging

**Fig. 3:**
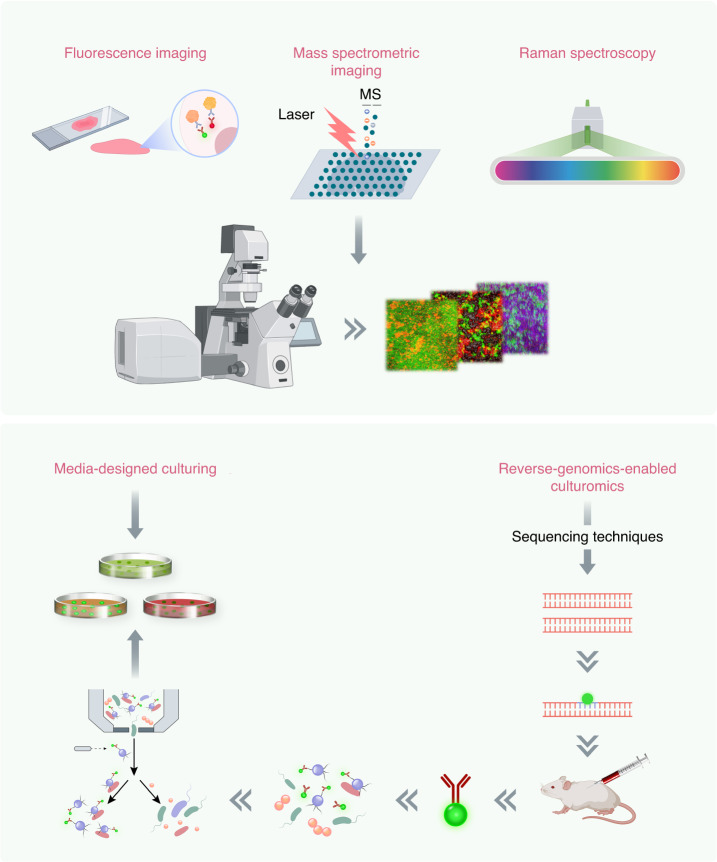
Imaging techniques and culturomics. Fluorescence techniques, mass spectrometry, and Raman spectroscopy have enabled the visualization of the spatial arrangement and interactions of oral microbiomes, providing valuable insights into the complex microbial communities within the oral cavity. Culturomics, on the other hand, plays a crucial role in the isolation and cultivation of novel and fastidious oral taxa. High-throughput approaches are employed to multiply and optimize culture conditions, enabling the recovery of specific taxa. While reverse-genomics-enabled cultureomics is a promising technique that can be used to cultivate novel lineages. This involves the identification of target microorganisms, prediction of highly expressed membrane proteins, synthesis of target-protein domain antigens, production of antibodies, labeling of target cells with fluorescent dye, sorting of cells based on antibody signal, and subsequent culturing

Microbial sequencing has revolutionized the qualitative and quantitative profiling of commensal and pathogenic microbes, providing valuable insights for disease diagnosis, prevention, and treatment.^41^ However, despite its precision and efficiency, sequencing has inherent limitations in dynamically and visually tracking target microflora, leading to incomplete knowledge of crucial microbial distribution and activity within the microbiota.^42^ To gain a comprehensive understanding of the intricate physiological and pathological processes of oral commensals and pathogens, more advanced methodologies and instruments are imperative. Among these, imaging techniques suitable for investigating this ecosystem across diverse contexts are highly desirable.^10,43^ Imaging can effectively delineate the spatial distribution and biogeography of commensal, pathogenic, or opportunistic oral bacteria. Additionally, imaging can provide critical insights into bacterial viability, metabolic exchanges, and host-microbe immune interactions.^44,45^ Broadly, the imaging modalities employed for oral bacteria include fluorescence techniques, mass spectrometry, and Raman spectroscopy (Fig. 3).

### The fluorescence imaging techniques

The utilization of the fluorescence imaging technique is effective in accurately identifying the location of target bacteria in the oral microbiota.^43^ The categorization of fluorescence imaging technology encompasses four distinct types, including fluorescent antibiotic-based staining, metabolic labeling facilitated visualization, nucleic acid staining, and genetic engineering-based imaging.

#### Fluorescent antibiotic-based staining

Certain antibiotics operate through the direct binding to a particular surface structure of bacteria, thereby preventing the possibility of employing these drugs as staining probes for bacteria.^46^ Fluorescently modified antibiotics have been employed in the monitoring of various bacterial species both in vitro and in vivo.

Vancomycin (Vanco) is a frequently employed antibiotic that specifically targets Gram-positive bacteria by impeding the synthesis of their cell walls through high-affinity binding to the D-Ala-D-Ala motif in peptidoglycan (PGN).^47^ This antibiotic has demonstrated potential as a fluorescent probe. It is noteworthy that Gram-negative bacteria are distinguished by the presence of lipopolysaccharides (LPS) in their outer membrane.^46^ A Gram-negative-specific fluorescent probe was developed by exploiting polymyxin B (PxB), a Gram-negative-specific antibiotic that binds to the lipid A of LPS.^48^ The PxB-based fluorescent probe, in combination with a Vanco-based probe, distinctly labeled two bacterial groups in the oral microbiota collected from a volunteer’s mouth, revealing significant phenotypical diversity within both groups.^46^

Furthermore, a Gram-negative-specific staining probe utilizing tridecaptin A, a novel class of antibiotics that selectively binds to the lipid II structure of Gram-negative bacteria has been developed.^49^ Upon combining tridecaptin A with the Vanco-based probe for labeling human oral microbiota obtained from the tongue dorsum, the Gram-negative and Gram-positive bacteria were differentially stained by the two probes, revealing significant phenotypic variations.^50^

#### Metabolic labeling-facilitated visualization

The metabolic labeling method is a conventional approach employed to investigate microbial metabolism and function. This technique involves the utilization of fluorescent probes to label the substances involved in the process of microbial metabolism, including sugars, amino acids, and nucleic acids, which are integral to the endogenous biosynthesis process.^51^ Subsequently, the biological material markers are naturally integrated into the microorganism’s structure through metabolic activity, thereby enabling the metabolic tagging function to be achieved.^43^ The utilization of this approach involves the fluorescent labeling of particular pathogenic bacteria or the commensal microbiota, to monitor their colonization or dissemination within the host. Metabolic labeling represents an indispensable technique for investigating bacterial activities in vivo and serves as a crucial strategy for visualizing oral bacteria that are not amenable to genetic manipulations.^52^ The covalent attachment of signaling tags to specific bacterial structures enables further functional examination of the interactions between the labeled macromolecules and the host.^10^

The regulation of bacterial growth is governed by the PGN cell wall, which is an essential and rigid structure consisting of glycan strands that are cross-linked by short peptides containing D-amino acid (DAA).^53^ To facilitate the real-time synthesis and detection of fluorescently labeled DAA components during cell wall formation, a chemical biology approach has been devised that can be applied across a diverse range of bacterial species and in live cells.^54^ Jiang et al. have successfully conjugated aggregation-induced emission luminous micelles with DAA, resulting in enhanced fluorescence that facilitates the fluorescent labeling of probiotics *Lactococcus lactis*. This approach holds potential for the non-invasive monitoring of ex vivo fluorescently labeled microbiota in deep tissues with high resolution.^11^

The majority of Gram-negative bacteria possess LPS that comprises a monosaccharide constituent, namely 3-deoxy-D-mannooctulosonic acid (Kdo), which is not present in Gram-positive bacteria or animals. In various Gram-negative species, the metabolic labeling of LPS has been demonstrated using an azido analog of Kdo, namely 8-azido-8-deoxy-Kdo.^55^ Hudak et al. have presented an interdisciplinary methodology that employs metabolic labeling in conjunction with bioorthogonal click chemistry, a technique performed within living organisms, to selectively label up to three major surface immunomodulatory macromolecules (PGN, LPS, and capsular polysaccharide) either concurrently or individually in live anaerobic commensal bacteria.^43^ This approach has facilitated the visualization and monitoring of the trajectory of labeled surface molecules from viable, luminal bacteria into distinct intestinal immune cells, which also sets the stage for observing the interactions between oral bacteria and host oral cells.^43^

#### Nucleic acid-based bacterial staining

Nucleic acid labeling, a conventional technique for staining diverse microbiological samples, employs either fluorogenic dyes that exhibit a strong binding affinity to RNA and DNA, or fluorophore-tagged DNA probes that selectively target bacterial rRNA.^56^ 4′,6-Diamidino-2-phenylindole (DAPI) is a commonly utilized nucleic acid labeling agent in oral microbiomes due to its notable affinity for nucleic acids and user-friendly nature.^57^

The fluorescence in situ hybridization (FISH) technique utilizes a fluorescent DNA probe with a significant level of sequence complementarity to selectively target the bacterial 16S rRNA.^58^ FISH has found extensive application in the identification of particular phylogenetically defined microorganisms within intricate environments, such as the oral microbiota.^56,58^ This methodology possesses the potential to be utilized in conjunction with confocal laser scanning microscopy (CLSM) to achieve precise reconstruction of the spatial organization of microbial communities within their respective habitats.^59^ Standard FISH protocols commonly utilize paraformaldehyde for chemical cross-linking or fixation, as well as ethanol, to facilitate probe penetration. These procedures lead to chemical alterations of nucleic acids and cellular demise, thereby impeding the utility of FISH, particularly in studying living cells.^60^ Batani et al. have addressed this issue by showcasing the successful introduction of labeled DNA probes into living bacterial cells through chemical transformation, leading to specific hybridization.^60^ The live-FISH technique thus eliminates the need for intricate probe design, rendering it a simple and promising method for the integration with other single-cell techniques technologies and for the isolation and culture of new microorganisms.

Borisy’s group proposed a CLASI-FISH (combinatorial labeling and spectral imaging) strategy to address the issue of the limited number of FISH probes that can be utilized on a single sample.^61^ This approach enables simultaneous labeling and accurate differentiation of a greater number of bacterial taxa. Additionally, Shi et al. introduced the high-phylogenetic-resolution FISH (HiPR-FISH) technique, which can identify hundreds of microbial species in complex communities. The utilization of a HiPR-FISH panel comprising 233 probes that specifically target 54 bacterial genera, in the analysis of plaque biofilms obtained from a healthy donor, revealed the presence of corn-cob-like spatial architectures within the human oral plaque microbiome.^56,62^

#### Genetic engineering-based imaging

Genetic engineering represents an elegant solution to the limitations of chemical labeling, such as the potential cytotoxicity of dyes and their dilution. This approach endows bacteria with imageable proteins that are expressed either inductively or spontaneously, such as fluorescence proteins and gas vesicle proteins.^42^ Genetic engineering with reporter genes, which predominantly express fluorescent proteins or luciferase, has emerged as the most extensively employed imaging modality in biological research.^63^ This technique has also found widespread application in visualizing transplanted bacteria in the gastrointestinal tract, and further development is needed for its application to the oral microbiome.

As a representative example of fluorescent proteins, the green fluorescent protein (GFP) offers a simple yet robust method for genetically labeling proteins of interest in bacteria. Despite the extensive research on GFP in oral bacteria, fluorescent protein probes exhibit several inherent limitations, including the potential for protein dysfunction resulting from the fusion process and the inability to function in anaerobic conditions due to their exclusive emission in the presence of molecular oxygen.^10^ Cao et al. have successfully encoded bacteria with a fluorescence-activating and absorption-shifting tag (FAST), which is a reversible on-off and emission switchable fluorescence. This development has enabled the creation of living bacterial probes for real-time dynamic and dual-modal imaging in both aerobic and anaerobic environments.^64^ The efficacy of this technique has been further validated in two murine models of gut and tumor localization.^42^ Which offers an alternative approach to studying the interaction between the microbiota and its host, through fluorescent imaging.

### Mass spectrometric imaging

Mass spectrometry imaging (MSI) is an analytical technique used to spatially detect and identify molecules in cells, tissues, or organisms. It employs an analytical probe, such as an ion beam, laser, or solvent junction, for in situ chemical desorption and/or ionization.^65^ MSI has gained popularity in the life sciences, particularly in microbiology, due to its ability to simultaneously detect biomolecules and their spatial distribution in complex samples. Among MSI techniques, matrix-assisted laser desorption/ionization mass spectrometry imaging (MALDI-MSI) is the most widely used for molecular imaging. Numerous studies have demonstrated the effectiveness of MALDI-MSI in studying microbial systems.^66,67^

In a hospital setting, MALDI is used to identify microorganisms in biological isolates and samples.^68^ This allows for rapid and accurate identification of species, subspecies, and strain levels in microbial communities within minutes.^69^ MSI has also been used to identify microbial biomarkers for diagnostic purposes. These pioneering investigations highlight the potential of MALDI-MSI in promptly and impartially identifying and diagnosing biomarkers of pathogenic microorganisms, including human papillomavirus and other pathogens.^70^

MALDI-MSI has also been integrated with various separation and imaging techniques, including scanning electron microscopy (SEM) to visualize microorganisms in rat lungs.^71,72^ The combination of MALDI-MSI and FISH microscopy is a powerful approach for visualizing metabolic phenotypes and studying microbial interactions in their natural environment.^73^ Future modifications of this technique may enable the integration of metabolic profiling with gene expression.^66,74^ MSI has also been used to investigate host-pathogen interactions and gain insight into biomolecular metabolism involved in microbial pathogenesis in vivo.^75^ Integration of chemo-histo-tomography with MALDI-MSI and micro-computed tomography has created a three-dimensional map of the chemical and physical interactions between microorganisms, such as bacteria and parasitic nematodes, and their host.^76^

Microbial ecosystems involve the secretion of various molecules, such as metabolites, lipids, polysaccharides, and peptides, through quorum-sensing mechanisms in response to environmental stress, nutrient availability, and territorial expansion.^77^ MADLI has been used to monitor the production of N-acyl-homoserine lactones, a type of quorum-sensing metabolite, during the development of *Pseudomonas putida* biofilm.^78^ This study revealed the correlation between biofilm development and the precise temporal regulation and spatial distribution of specific quorum-sensing metabolites in *P. putida*. Laser-post-ionization modules integrated into mass spectrometers have significantly enhanced the analytical sensitivity, allowing for the examination of metabolic exchange between competing microorganisms cultivated on polyamide membranes.^79^ This research provides deeper insights into the complex chemical communication pathways of microorganisms.^79,80^

### Raman spectroscopy

Raman spectroscopy (RS) is a technique that utilizes inelastic scattering spectra based on the Raman effect, and signals were obtained through the use of scattered light with a different frequency from the incident light.^81^ This method offers fast, accurate, and sensitive in situ detection analysis. RS is capable of sensitively and accurately reflecting changes in material composition and structure, making it a valuable tool in medical detection across various fields. For instance, RS has been utilized to differentiate between bacteria or between neoplastic and normal brain tissues, aiding in diagnosis and prognosis evaluation.^82,83^

Numerous oral diseases are associated with oral microbial dysbiosis, resulting in distinct differences in essential components between their lesions and normal tissues. Consequently, alterations in bacterial composition and the constituents of saliva and tissue can serve as distinctive markers for these oral diseases, with RS being recognized as a valuable diagnostic tool.^82^ The utilization of RS has been shown to enable the identification of metabolic variances thereby contributing to the identification of different biofilms. In Gieroba et al.’s research, Raman spectra were obtained and analyzed from individual biofilms of *S. mutans*, *Streptococcus sanguinis*, and *Streptococcus sobrinus.*^84^ The most significant differences were observed in the spectral region corresponding to lipids, amides, and carbohydrates, which were indicative of the respective biological and cariogenic characteristics. The exposure of cariogenic biofilms to quaternary ammonium resulted in a significant reduction in the intensity of the 484 cm^−1^ peak in the Raman spectra, which is indicative of polysaccharides or carbohydrates.^85^ The observed changes were found to be concentration-dependent and time-dependent, thus highlighting the potential of Raman spectra as a tool for evaluating the efficacy of anti-caries drugs.

Furthermore, the capability of RS to differentiate various subgingival bacteria has been demonstrated through the utilization of in situ Raman microprobe spectroscopy. This method was employed to monitor the metabolic alterations of *Porphyromonas gingivalis* on bioceramic surfaces coated with the antibacterial agent silicon nitride, ultimately revealing the production of peroxynitrite in *P. gingivalis.*^86^ In the in vitro research on subgingival biofilm models, Kriem et al. utilized Confocal Raman Microscopy in conjunction with two-way orthogonal Partial Least Square with Discriminant Analysis.^87^ This approach was applied to the analysis of various oral subgingival species, as well as artificial subgingival mono-species biofilm models. The method demonstrated the ability to analyze and predict the identity of both planktonic and mono-species biofilm species, indicating its potential as a technique for mapping oral multi-species biofilm models.^87,88^ Based on previous techniques, a standard Raman spectral detection process was developed to distinguish between various serotypes of *P. gingivalis*, *Aggregatibacter actinomycetemcomitans*, and *Streptococcus* spp.^89^

## Culturomics

The “omics” era has significantly advanced our understanding of the identity and potential functions of oral microorganisms, and imaging techniques have enabled the visualization of oral microbiota. Despite the wealth of knowledge about oral microbiomes gained from genome-driven discovery, it remains imperative to isolate and cultivate species from these unexplored lineages to validate genome-based predictions regarding their cellular biology, physiology, and ecological functions. Culturomics is a culture methodology characterized by a high-throughput approach that combines various selective and enriched culture conditions with identification techniques, including matrix-assisted laser desorption ionization-time of flight mass spectrometry (MALDI-TOF MS) and 16S rRNA gene sequencing.^12^ The emergence of culturomics as a field has facilitated the retrieval of microbes that were previously deemed “difficult to culture”. Recent technological advancements have spurred the recovery of low abundance and/or novel lineages of microbes, resulting in a significant proportion of microbes being recovered from various biomes that were previously considered uninhabitable by microorganisms^90,91^ (Fig. 3).

While Cultureomics has primarily been utilized in the examination of intestinal bacteria, there have been limited investigations into its application in the realm of oral microbiology. Martellacci et al.’s research represents the first instance of culturomics being employed in the field of dentistry.^92^ Their study yielded the isolation of 48 bacterial species from sub-gingival and peri-implant plaques, 12 of which had not previously been associated with the oral cavity and 5 of which had never been isolated from clinical specimens.^92^ Additionally, Wang et al. utilized Cultureomics technology to isolate bacteria from oral samples, resulting in the identification of 144 strains, including 5 potential new species.^93^ The results indicate that culturomics provides a comprehensive and precise descriptive analysis of the oral microbiota, rendering it a viable approach for tailoring the diagnosis and treatment of oral ailments.

Cross et al.’ study demonstrated the use of genomic information to directly facilitate the physical isolation and cultivation of target organisms, known as “targeted reverse genomics”.^6^ This approach involves identifying genes in the uncultured target organism’s genome that encode membrane proteins with extracellular domains. Antibodies raised against these domains can then be used to label oral samples containing the target organisms, allowing for their isolation and cultivation. This method has successfully led to the isolation of novel human oral TM7 bacteria and has made progress toward the cultivation of an oral SR1 bacterium. Targeted reverse genomic-based cultivation has the potential to greatly impact oral microbiology by enabling the isolation and characterization of many previously unknown lineages.

## Conclusion

Elucidating the structure and function of oral microbiomes is crucial for understanding human health and disease. Recent advancements in technology and methodology have revolutionized the characterization of oral bacteria, shifting from a descriptive approach to a dynamic understanding of genes, transcripts, proteins, metabolites, and their interconnected activities. Sequencing techniques have rapidly evolved to enable high-resolution analysis of oral ecosystems at a multi-omic level. Novel imaging modalities allow for the identification of microbial identity, function, and localization within intact communities at an increasingly precise spatial and chemical resolution. Culturomics has expanded the cultivation of previously fastidious oral bacteria, facilitating experimental validation of predicted characteristics. Synthetic approaches enable the intentional manipulation of microbial ecologies to dissect the principles governing their activities and interactions with the host and other microorganisms. Integrative microfluidic models and organ chips replicate the oral interfaces, providing a platform for mechanistic studies. However, there are still significant knowledge gaps regarding community assembly, metabolism, microbe-microbe/host signaling, circadian rhythms, stress response dynamics, and pathobiology. Key questions remain unanswered regarding developmental origins, assembly principles, triggers of homeostasis versus dysbiosis, functional biomarkers of disease, and strategies for deliberate modulation of oral ecosystems. Although modern omics techniques provide a comprehensive list of components, linking genotypes and molecular data to community physiologies and host interactions remains challenging. Temporally tracking community dynamics and activities in their natural environment continues to present obstacles. There are significant translational opportunities to leverage emerging insights for more precise and personalized diagnostics and therapeutics targeting the oral microbiome. Future progress requires an integrated framework that synergistically combines cultivation, omics, imaging, biomaterials, microfluidics, bioreactors, animal models, and computational analysis from a systems perspective. Extensive data integration platforms and artificial intelligence will be essential in contextualizing complex multimodal datasets. Expanding genomic and phenotypic characterization of cultivatable oral taxa through culture collections will enhance the functional interpretation and validation of metagenomic discoveries. The synergy between metagenomics, metatranscriptomics, metaproteomics, and metabolomics will unravel the molecular pathways that connect genetic potential to community physiologies. Spatial transcriptomics and proteomics will map functional activities to specific habitats and niches. Spectroscopic techniques will provide signatures of community state transitions from health to disease. Biomaterial and microfluidic models will recreate spatiophysical gradients and biogeography. Machine learning will integrate diverse data streams to identify predictive biomarkers and generate biological insights. In summary, the convergence of innovative approaches for multi-view characterization holds the promise of uncovering the principles governing the emergence of oral microbiome developmental trajectories, assembly, microbe-microbe interactions, cooperativity, virulence, and pathobiology within the context of human holobiont physiology. These exciting advances foreshadow a transformation from cataloging components to gaining illuminative insights into the complex processes orchestrating oral microbial ecology. Harnessing this knowledge offers immense potential for predicting, preventing, diagnosing, and treating microbiome-associated oral diseases. We stand on the cusp of a new era where mechanistic oral microbiology paves the way for precise and personalized therapies targeting the microbiome to enhance human health.
